# MSH6 and PMS2 expression in colorectal carcinoma

**DOI:** 10.6026/973206300214968

**Published:** 2025-12-15

**Authors:** Safin M, Marylilly S, Janhavi MS

**Affiliations:** 1 Department of Pathology, Sree Balaji Medical College and Hospital, Chennai-44, Tamilnadu, India

**Keywords:** microsatellite instability, MSH6, PMS2, colorectal carcinoma, immunohistochemistry

## Abstract

Microsatellite instability (MSI) is a key feature in colorectal carcinomas (CRCs), but its role in diagnosis and prognosis, particularly through immunohistochemical markers like MSH6 and PMS2, remains underexplored. Conducted at Sree Balaji Medical College and Hospital, 50 histologically confirmed CRC samples were analyzed. Combined loss of MSH6 and PMS2 (MSI-H) was found in 30% of cases, particularly in poorly differentiated tumors, T3 stage tumors, stage II and III cancers, and node-positive groups. Thus, understanding of microsatellite instability (MSI) detection in colorectal carcinomas (CRCs) using immune histochemical markers MSH6 and PMS2, improving diagnostic and prognostic approaches for CRC.

## Background:

Colorectal cancer is one of the most common and deadly cancers worldwide, with 1.93 million new cases and about 9.3% of cancer deaths reported in 2022. In India, the incidence is 4.9 and mortality 2.9 per 100,000 population, with men affected more often than women [1]. It represents a heterogeneous group of malignancies driven by genetic predisposition (e.g., Lynch syndrome, FAP), environmental exposures, lifestyle factors such as diets rich in red or processed meat, low-fibre diet, obesity, reduced physical activity, tobacco use, alcohol intake, gut microbiota alterations, and chronic inflammatory conditions like inflammatory bowel disease [2]. An important aspect of CRC pathogenesis involves epigenetic and genetic factors which can lead to mutations and chromosomal aberrations that fuel tumorigenesis includes -Chromosomal Instability (CIN), Microsatellite Instability (MSI) and CpG Island Methylator phenotype (CIMP) [3]. MSI occurs in about 15-20% of all colorectal cancers with 1-3% attributable to Lynch syndrome or HNPCC [3,4]. Among the various mechanisms underlying genetic instability, microsatellite instability has emerged as a critical feature of colorectal cancer, affecting both sporadic [75%] and hereditary cases [4]. Additionally, MSI can also occur in sporadic CRCs by epigenetic silencing of MMR genes, notably MLH1 via CpG island hypermethylation [3]. MSI refers to accumulation of mutations within short, repetitive DNA sequences called microsatellites, resulting from defects in DNA mismatch repair (MMR) system [3]. The DNA mismatch repair (MMR) system, involving proteins like MLH1, MSH2, MSH6, and PMS2, corrects replication errors and mutations in these proteins cause microsatellite instability (MSI) due to accumulated DNA errors [5]. MSH6 is part of the MSH2/MSH6 heterodimer, which primarily detects single base mismatches and small insertion-deletion loops, while PMS2 is a component of the MLH1/PMS2 complex, which functions in the repair process by recognizing the mismatched bases and initiating the repair cascade [3, 5]. While PCR-based molecular analysis of microsatellite markers has traditionally been used to detect MSI, it is often costly and time-consuming, whereas immunohistochemistry (IHC) has now become widely adopted, reliable, and economical alternative that enables direct visualization of MMR protein loss or expression in tumor tissues, making it a more practical approach for identifying MMR deficiency in routine clinical settings [6].

Among the MMR proteins, IHC with PMS2 and MSH6 is more diagnostically reliable, as it can reveal defects in their own genes as well as mutations in MLH1 and MSH2, whereas IHC with MLH1 and MSH2 may miss a subset of abnormalities [3]. MSI-H CRC usually shows poor differentiation, mucinous histology, lymphocytic infiltration, right-sided location and early-stage presentation, while in metastatic disease it is more common in women and the elderly, often with nodal or peritoneal rather than liver metastases. MSI-H tumors generally have favourable prognosis than MSS tumors, especially in stage I and II disease, but stage III cases with concurrent BRAF mutations may be associated with poorer outcomes [7]. MSI-H tumors known to have high tumor mutational burden (TMB), which may contribute to their responsiveness to immunotherapy, particularly immune checkpoint inhibitors like pembrolizumab [8]. Therefore, it is of interest to evaluate MSH6 and PMS2 expression by immunohistochemistry in colorectal carcinomas at a tertiary care centre to assess microsatellite instability and its correlation with clinicopathological parameters.

## Materials and Methods:

This cross-sectional study (2023-2025) was done in the Department of Pathology, Sree Balaji Medical College and Hospital, Chromepet, Chennai, by the approval of Institutional Human Ethical Committee. The study evaluated role of MSH6 and PMS2 immunohistochemical markers in detecting MSI in colorectal carcinoma. Cases included were histopathologically confirmed colorectal carcinomas, encompassing patients across all age groups, including those who had received chemotherapy or radiotherapy. Exclusion criteria were non-neoplastic or premalignant lesions (such as colitis, IBD, adenomas, and serrated lesions), non-representative or poorly processed tissues, specimens with extensive necrosis or haemorrhage, samples not received in formalin, and patients unfit for colorectal biopsy; faculty members and students were also excluded. Total of 50 formalin-fixed, paraffin-embedded (FFPE) tissue samples (36 resections and 14 biopsies) were processed and H & E stained for histopathological evaluation as per WHO criteria. Immunohistochemistry was performed on 3 µm sections using rabbit monoclonal antibodies MSH6 (Clone EP49) and PMS2 (Clone EP51), with detection by PolyExcel HRP/DAB and Harris haematoxylin counterstain. The study variables included demographic data (age, sex) and tumor characteristics (site, specimen type, histological grade, histological type, tumor stage, nodal stage and stage group, along with MSH6 and PMS2 expression assessed by immunohistochemistry.

## Microsatellite Instability (MSI) Categorization and scoring immunoreactivity:

The MSI status of the samples was classified into three categories: Microsatellite Stable (MSS), Microsatellite Instability-Low (MSI-L), and Microsatellite Instability-High (MSI-H).

[1] MSS - intact nuclear expression of all markers. 

[2] MSI-L - loss of nuclear expression of one of the markers.

[3] MSI-H - loss of nuclear expression of two or more markers.

[4] Undetermined or Indeterminate cases- improper staining of the markers.

Immunoreactivity was evaluated based on nuclear staining in neoplastic glands,

[1] Loss of nuclear expression in tumor cells.

[2] Intact nuclear expression in tumor cells - intensity of positive nuclear staining not recommended in determining the MSI.

[3] Undetermined or Indeterminate - poor nuclear expression of neoplastic cells as well as poor internal control expression.

For our study, we used internal positive control - Positive nuclear expression in normal colonic mucosa, inflammatory cells predominantly lymphocytes or centre of lymphoid follicles.

Since we use only MSH6 & PMS2 markers for our study, we categorized the results as following 

[1] Combined MSH6 & PMS2 markers positivity.

[2] Isolated MSH6/PMS2 marker loss.

[3] Combined MSH6 & PMS2 markers negativity.

[4] Indeterminate or undetermined.

## Statistical analysis:

Data were entered in Microsoft Excel and analysed using IBM SPSS Statistics for Windows, Version 29.0 (Armonk, NY: IBM Corp). Descriptive statistics were applied, with frequencies and percentages used for categorical variables and mean ± standard deviation for continuous variables. Chi-square test was employed to assess correlations between categorical variables, and p-value < 0.05 was considered statistically significant.

## Results:

Total of 50 CRC specimens, including both resected specimens and colonoscopy biopsies, were collected between 2023 and 2025. These specimens were analysed to evaluate nuclear expression of MSH6 & PMS2, categorized as combined MSH6 & PMS2 positivity, isolated marker loss and combined MSH6 & PMS2 loss and correlated with clinical parameters - age, gender, tumor site, histological type, histological grade, tumor stage and nodal status. Analysis of MSH6 & PMS2 expression in 50 samples showed combined markers loss in 15 cases, 8 cases show isolated marker loss, another 22 cases show combined markers positivity and 5 cases were indeterminate (Table 1 - see PDF & Figure 1 - see PDF, 2 - see PDF, 3 - see PDF). The mean age of patients was 55.9 ± 12.1 years (range 31-80), with the majority in the 61-70- year group (34%). Combined MSH6 and PMS2 loss was most frequent in this age group (40%), without significant correlation (p=0.406) with age. Of the 50 patients, 32 were male (64%) and 18 female (36%) with a male-to-female ratio of 1.7:1. Combined loss was seen in 8 males (53.3%) and 7 females (46.7%), with no significant correlation (p=0.714) with gender. Of the 50 cases, 42% arose in the right colon, 24% in the left colon, and 34% in the rectum. Female gender was significantly associated with right-sided tumors (p=0.001). Combined MSH6 & PMS2 loss was most frequent in right colon cancers (73.3%) with no significant correlation (p=0.063) was observed between tumor site and combined loss (Table 2 - see PDF). The most predominant histological type was adenocarcinoma NOS (72.0%), followed by adenocarcinoma with signet ring cell (16.0%), mucinous adenocarcinoma (6.0%) and undifferentiated carcinoma (6.0%). Combined MSH6 & PMS2 loss was observed in 6 out of 8 cases (40%) diagnosed as adenocarcinoma with signet ring cells, followed by 5 out of 36 cases (33.3%) diagnosed as adenocarcinoma NOS. A statistically significant correlation (p =0.027) noted between histological type-specifically signet ring cell type, adenocarcinoma NOS and combined markers loss in CRCs. Among histological grade, most common were moderately differentiated (G2) accounts for 25 cases [50.0%], followed by well differentiated (G1) accounts for 14 cases (28.0%), and poorly differentiated (G3) 11 cases (22%). Combined MSH6 & PMS2 loss observed 8 out of 11 poorly differentiated cases (53.3%), followed by moderately differentiated (26%.7), and well-differentiated cases (20%). 

Despite these variations, the correlation between histological grade and combined markers loss was statistically significant (p = 0.038), with loss being predominantly observed in poorly differentiated colorectal cancers. Out of 36 resected specimens [biopsies excluded]; Tumor staging was distributed as T2 (12 cases), T3 (14 cases), T4 (5 cases), and YpT2/3 (5 cases). Combined marker loss was predominantly seen in advanced stages, with the highest frequency in T3 tumors (8 cases, 72.7%), followed by T4 tumors (2 cases, 18.2%). This association demonstrated high statistical significance (p=0.0005), indicating that combined MSH6 & PMS2 loss strongly correlates with advanced T stage in CRCs. Out of 36 resected specimens [biopsies excluded]; Nodal stage was distributed as 19 cases of node-negative groups NX+N0 (52.8%)) and 17 cases of node-positive groups N1+N2 (47.2%)). Combined marker loss was noted predominantly in node-positive groups of 6 cases (54.5%), followed by node-negative groups of 5 cases (45.5%). Thus, although combined MSH6 & PMS2 loss was seen in both node- negative as well as positive groups, predominance of loss in node-positive cases highlights a significant association between nodal status and combined MSH6 & PMS2 loss in CRCs (p=0.004).In our study, out of 36 resected specimens [biopsies excluded]; 6 cases diagnosed stage group I (16.7%), 9 cases diagnosed stage II (25.0%), 16 cases diagnosed stage III (44.4%) as well as 5 cases (chemotherapy treated) Stage y II, III (13.9%). Combined MSH6 & PMS2 loss frequently observed in stage III, 6 out of 16 cases (54.5%), followed by 5 out of 9 cases in stage II (45.5%). The correlation between stage and combined marker loss was highly statistically significant [p=0.0005], indicating strong correlation with stage III and II CRCs.

## Discussion:

According to the recent CAP (College of American Pathologists) protocol version 1.3.0.0, MSI status is classified as follows: MSS, where all markers show nuclear positivity; MSI-L, where one marker exhibits full loss of expression and MSI-H, where two or more markers show full loss of expression [9]. In this study, we focused on evaluating the loss of MSH6 and PMS2 nuclear expression as a means of identifying MSI in CRC. We categorized the samples into three groups: combined MSH6 and PMS2 positivity, isolated marker loss, combined MSH6 and PMS2 loss (MSI-High) and indeterminate category. Indeterminate cases through IHC should ideally be further evaluated using molecular methods such as PCR-based MSI testing or next-generation sequencing (NGS) to allow proper categorization. The results were then correlated with age, gender, tumor site, histological grade, histological type, tumor stage, nodal stage and stage group. In our study, we collected 50 colorectal carcinoma specimens, including 36 resected and 14 biopsy samples. The average age of patients was 55.9±12.1 years, with most common age group being 61-70 years (34.0%), followed by 51-60 years (24.0%). Jiachen Zhang *et al.* [10] reported that majority of CRC cases were in 61-70 years age group (35.7%). Our study, highest percentage of combined MSH6 & PMS2 loss (40%) was observed in 61-70 year age group, followed by 26.7% in 41-50 year group and was not statistically significant. Sanghee Kang *et al.* [11], studied that MSI-H (50%) was noted in cases taken in age group of <70 years as well as >70 years showed no statistical significance.

Regarding gender distribution, our study found a male predominance in CRC cases, with male-to-female ratio of 1.7:1. Zhang *et al.* [10] studied MSI with male preponderance in CRC cases. The combined MSH6 & PMS2 loss observed in 46.7% of females and 53.3% of males, showing no statistically significant correlation. Catherine Gutierrez *et al.* [12], reported high prevalence of MSI-H in females (18.2%) compared to males (13.8%) with statistical significance [p=0.001]. However, difference in MSI-H prevalence between genders may be attributed to variations in the study sample population. We noted statistically significant correlation [p=0.001] of gender and tumor site, revealing most of the right colon cancers (66.7%) are predominant in female patients and rectal cancers (82.4%) are predominant in male patients. Schmuck *et al.* [13], reported female predominance of right colon cancer [45%] with high statistical significance [p<0.001]. Our study also found that the right colon was the most common site for MSI-H CRCs. Although, 73.3% of right colon cases showed combined MSH6 & PMS2 loss but no statistically significant correlation obtained (p=0.063) in our study. This result is consistent with other studies, such as Lin Yuan *et al.* [14], who observed a higher frequency of MSI-H in right colon tumors, followed by left colon and rectal tumors. Additionally, Kang *et al.* [11] and Nayak *et al.* [15], also reported that MSI-H was frequently noted in right colon cancers. This might be clinically important as right colon cancers are often associated with MSI-H and also have an impact on prognosis as well as therapeutic strategies. Among histological grade, our study demonstrated statistically significant correlation (p=0.038) between combined MSH6 & PMS2 loss with histological grade, predominance observed in (53.3%) poorly differentiated tumors followed by moderately differentiated tumors. This aligns with results of Garcia *et al.* [16], studied showed that MSI-H was highly noted in tumors with poor differentiation of statistical significance (p=0.015). Among histological type, Combined MSH6 & PMS2 loss was most frequently observed in adenocarcinoma with signet ring cell type (40%), followed by adenocarcinoma NOS (33.3%). This distribution showed a statistically significant correlation (p = 0.027), indicating that combined marker loss is significantly associated with specific histological subtypes, particularly signet ring cell type and adenocarcinoma NOS in CRCs. This aligns with results of Garcia *et al.* [16], reported MSI-H in mucinous and/or signet ring differentiation with statistical significance (p = 0.016).

Gutierrez *et al.* [12], reported higher rate of MSI-H in medullary carcinoma (41.1%), followed by mucinous adenocarcinomas (24.6%). Regarding Tumor stage, combined MSH6 & PMS2 loss was predominantly seen in advanced stages, with the highest frequency in T3 tumors (72.7%), followed by T4 tumors (18.2%). This association demonstrated high statistical significance (p=0.0005), indicating that combined MSH6 & PMS2 loss strongly correlates with advanced T stage in CRCs. This is consistent with Karavin *et al.* [17], who studied that MSI- H was highly noted in T3 (88%) and T4 (11%) with statistical significance (p= 0.014). Lymph node positive cases were significantly associated [p=0.004] with combined MSH6 and PMS2 loss [54.5%], followed by node-negative groups of 5 cases (45.5%). in our study out of 36 resected cases. Ismael *et al.* [18], studied that MSI- H in Egyptian patients and reported that MSI-H was noted in both N0 and NI/N2 [15.4% each] equally with no statistical significance. However, contrary to our findings, Yuan *et al.* [14] and Mohan *et al.* [19], reported MSI-H predominantly in tumors with negative nodal involvement or early stage I/II. The discrepancy could be attributed to differences in sample populations and study methodologies. Regarding stage group, our study out of 36 resected cases revealed that combined MSH6 & PMS2 loss prevalent in stage III tumors (54.5%), followed by stage II tumors (45.5%), with a highly significant correlation combined markers loss between and III/II stages (p=0.0005). In contrast, Yuan *et al.* [14] and Merok *et al.* [20] reported MSI-H in I/II stages than stage III of CRCs. In our study, although stage III cases constituted the majority, both stage II and stage III demonstrated combined MSH6 & PMS2 loss, which may have influenced the overall distribution of MSI across the stages. In our study, MSI-H was detected in 15 out of 50 cases (30%), which is little above prevalence around 15-20% MSI of all colorectal cancers. This can be explained by the clinicopathological profile of our study, which included high proportion of right colon tumors as well as Stage II/III cases, both known to show increased MSI-H frequency. Hospital-based selection may also have introduced referral or sampling bias, enriching this study for MSI-positive tumors. Additionally, regional and ethnic variations could contribute to this finding. Nonetheless, limited sample size and single-institution design restrict generalizability of our findings, highlighting need for larger, population-based studies to validate these results.

## Conclusion:

The importance of MSH6 and PMS2 as immunohistochemical markers for identifying microsatellite instability in colorectal carcinoma is shown. We reported significant association between combined marker loss with parameters such as right-sided colon tumors, poorly differentiated, signet ring cell type, advanced tumor stages, positive lymph node and stage group III, II, reinforcing clinical importance of MSI testing in CRCs. However, further research with larger sample sizes and more diverse cohorts is necessary to better understand the diagnostic and prognostic significance of MSI and its role in guiding treatment decisions. Integrating MSI testing into standard clinical practice has the potential to enhance prognostic precision and guide more personalized therapeutic strategies for CRC patients.
